# Nosocomial outbreak of the pandemic Influenza A (H1N1) 2009 in critical hematologic patients during seasonal influenza 2010-2011: detection of oseltamivir resistant variant viruses

**DOI:** 10.1186/1471-2334-13-127

**Published:** 2013-03-07

**Authors:** Caterina P Pollara, Giorgio Piccinelli, Giuseppe Rossi, Chiara Cattaneo, Francesca Perandin, Silvia Corbellini, Dolores De Tomasi, Carlo Bonfanti

**Affiliations:** 1 Laboratory of Microbiology and Virology, A. O. Spedali Civili, Brescia, University of Brescia, Brescia, Italy; 2 Department of Haematology, A. O. Spedali Civili, Brescia, Italy; 3 Institute of Microbiology, A. O. Spedali Civili, Brescia, University of Brescia, P.le Spedali Civili, 1 25123 Brescia, Italy

**Keywords:** Pandemic, H1N1, Oseltamivir, Resistance

## Abstract

**Background:**

The pandemic influenza A (H1N1) 2009 (H1N1pdm09) virus infection caused illness and death among people worldwide, particularly in hematologic/oncologic patients because influenza infected individuals can shed virus for prolonged periods, thus increasing the chances for the development of drug-resistant strains such as oseltamivir-resistant (OST-r) variant.

**Methods:**

The aim of our study was to retrospectively evaluate the clinical importance of OST-r variant in circulating strains of the pandemic H1N1pdm09 virus. By means of RT-PCR and Sanger sequencing we analysed the presence of OST-r variant in 76 H1N1pdm09 laboratory-confirmed cases, hospitalized at the hematologic/oncologic ward at Spedali Civili of Brescia –Italy.

**Results:**

Out of 76 hospitalized hematologic/oncologic patients, 23 patients (30.2%) were infected by H1N1pdm09 virus. Further investigation revealed that 3 patients were positive for the OST-r variant carrying the H275Y mutation. All the 23 infected patients were immuno-compromised, and were under treatment or had been treated previously with oseltamivir. Three patients died (13%) after admission to intensive care unit and only one of them developed H275Y mutation.

**Conclusions:**

Our retrospective observational study shows that pandemic influenza A (H1N1) 2009 virus can cause significant morbidity and even mortality in hematologic/oncologic patients and confirms the high rate of nosocomial transmission of pandemic H1N1pdm09 virus in these critical subjects. Indeed, the reduction in host defences in these hospitalized patients favoured the prolonged use of antiviral therapy and permitted the development of OST-r strain. Strategies as diagnostic vigilance, early isolation of patients and seasonal influenza A(H1N1) vaccination may prevent transmission of influenza in high risk individuals.

## Background

The pandemic influenza A (H1N1) 2009 virus (H1N1pdm09) broke out in Mexico and USA during the late spring and early summer of 2009. Due to its rapid widespread transmission, H1N1pdm09 virus was declared as novel influenza pandemic virus in June 2009 by the World Health Organization (WHO) [1-6]. Indeed the genomic analysis of this new 2009 influenza virus indicated a genetic viral reassortment and it contained genes of influenza A virus strains that are endemic in swine, avian and human species [7,8].

The 2009 pandemic virus was found susceptible to neuraminidase inhibitors (NAI) antiviral drugs such as oseltamivir (Tamiflu TM) and zanamivir (Relenza TM) that have been used extensively for chemoprophylaxis and treatment in 2009 pandemic virus. The emergence of oseltamivir-resistant (OST-r) pandemic H1N1pdm09 variant, characterized by the single amino acid change H275Y in the neuraminidase (NA) glycoprotein [9-13], was detected in many countries [14], especially in Italy [15-18]. The OST-r variant has been detected in immuno-compromised patients that are at risk for serious complications from seasonal influenza [2,19-22]. The nosocomial transmission of H1N1pdm09 resistant variants may cause an increase of morbility and mortality in this group of subjects.

The aim of this study was to investigate nosocomial viral transmission of H1N1pdm09 in a cluster of critical hematologic patients and the clinical importance and impact of OST-r variant viruses.

## Methods

### Study population

In January 2011, an outbreak of H1N1pdm09 occurred in a Hematology ward at Spedali Civili of Brescia (Italy). Respiratory specimens from symptomatic contact patients of the presumed index patient were analyzed by reverse transcriptase-polymerase chain reaction (RT-PCR) for influenza A/B virus. Screening was performed on 134 specimens obtained from 76 hospitalized patients (age range: 23-76 years). H1N1pdm09 positive samples were further tested for OST-r variant.

In our laboratory, the current diagnostic algorithm for testing of Influenza includes primary screening using a multiplex commercial real-time RT-PCR assay (Flu A/Flu B Q-PCR Alert Kit, Nanogen Advanced Diagnostics, Italy) targeting the matrix gene of all human influenza A and B viruses. Positive samples were subsequently tested for H1N1pdm09 sub-type by a commercial assay (Fast set H1N1v-Arrowdiagnostics, Italy). H1N1pdm09 positive samples were further tested for oseltamivir resistance using an in house RT-PCR [23,24] and the results confirmed using Sanger sequencing method. We retrospectively obtained the clinical data of the confirmed cases and reviewed the medical records. All patients submitted to RT-PCR test for H1N1pdm09 presented influenza-like illness (ILI) symptoms, and the positive cases were administered oseltamivir therapy: 75 mg twice/ day, for five days.

Clinical samples (nasopharyngeal swabs, bronchoalveolar washings or respiratory secretions) were collected from all patients using a pernasal flocked swab and stored in a UTM-RT tube (Kit Cat. No. 360c, Copan Italia, Brescia, Italy). Each sample was immediatly tested by real-time PCR in order to identify A and B influenza viruses as previously described. Diagnosis of influenza was invariably provided within 8–24 hours after the receipt of the sample.

### Ethical considerations

The investigation of this outbreak did not involve any planned activity that could have been reviewed prospectively by an institutional review board or ethics committee. The Ethics commette was consulted retrospectively and agreed to the approach as described for reporting clinical information obtained during the investigation. All patients received study information and signed informed consent.

### RNA extraction, real-time RT-PCR , multiplex and one step RT-PCR

DNA/RNA was extracted by an automatic nucleic acid platform (Nuclisens EasyMag - Biomerieux). Aliquots of 400 μl of each respiratory sample were added to 1600 μl of lysis buffer and the mix was loaded in the instrument. After a lysis step (10 minutes at room temperature), 65 μl of magnetic silica, 10 μl of internal control (CPE, Nanogen Advanced Diagnostics S.R.L), purified beta globin sequence and 55 μl of wash reagent were added to each specimen. Total nucleic acid was automatically extracted, eluted in 110 μl of specific buffer and immediately processed by real time PCR. Aliquots were stored at -80°C.

Extracted viral RNA was tested by Flu A/Flu B Q-PCR Alert Kit (Nanogen Advanced Diagnostics, Italy), using ABI Prism 7300 Fast Real Time PCR System (Applied Biosystems, Italy). This test is based on simultaneous amplification of the matrix region of Influenza A and B and of the internal control human beta globin gene. Beta globin DNA was added to each sample to monitor the efficiency of extraction and amplification procedures. Specific TaqMan probes labelled with different reporter molecules were used to detect amplification of the targets.

The positive samples for influenza A virus were tested for H1N1pdm09. Viral RNA was tested by Fast set H1N1v (Arrowdiagnostics, Italy) using ABI Prism 7300 Fast Real Time PCR System (Applied Biosystems, Italy). This test is constituted combining a two-step reverse transcription-multiplex PCR with typing and sub-typing on the electronic microarray. This assay distinguishes influenza virus types A and B, and identifies common influenza virus A subtypes H1N1 and H3N2, as well as the potentially pandemic avian influenza virus subtype H5N1.

The H1N1 positive samples were tested for OST-r variants by one step RT-PCR amplification using single nucleotide polymorphism (SNP) and directly sequenced by cycle sequencing. This one step RT-PCR amplification was made in house following S. Wong’s protocol [23]. Amplification was carried out using SuperScript TM One-Step Quantitative Kit (Invitrogen) in a final volume of 25 μl, with 5 μl of extracted RNA as template, 800 nM each of the forward and reverse primers and 200 nM of each probe. The amplification conditions were: reverse transcription at 48°C for 30 min, Taq activation for 10 min at 95°C, followed by 45 cycles of amplification comprising denaturation for 15 sec at 95°C, annealing and primer extension for 1 min at 60°C.

### Sequencing of NA gene

The 2009 influenza A(H1N1) NA gene was amplified directly from clinical specimen using the Superscript III One-step RT-PCR amplification kit (Invitrogen, Carlsbad, USA).

After RNA extraction, a 415 bp fragment of the NA gene was amplified using the following primers: forward AACACAAGAGTCTGAATGTGC, reverse ACCGTTTCTTGAACTAATGCTT [23]. The PCR products were further purified using QIAquick® Kit (Qiagen, Italy) and sequenced using Big Dye® Terminator v3.1 Cycle Sequencing Kit (Applied Biosystem, Italy) with an ABI PRISM 310 Genetic Analyzer. Manual examination of the electropherogram was also performed for the identification of mixed bases, following in-house protocols for diagnosis confirmation (protocols available upon request).

## Results

During the period January 1^st^ to March 31^st^ 2011, in the Hematology ward of Spedali Civili of Brescia (Italy), 23 patients out of 76 (30.2%) developed nosocomial H1N1pdm09 from the likely index case. A total of 134 specimens were performed: 41 samples resulted positive for H1N1pdm09, and subsequently were tested for oseltamivir resistance and 3 samples were positive. Nosocomial acquisition was defined as the development of symptoms attributable to pandemic influenza A that was not present at admission in the hospital but set within 48 hours. Most patients exhibited fever (92%) and cough (90%); dyspnoea, sore throat and rhinorrhea were reported in 45% of the patients. Three cases of OST-r variant were identified during the peak activity of H1N1pdm09 infection and in the same period three patients who required transfer to the intensive care (ICU) because of their critical respiratory status died for fatal influenza virus pneumonia (13.1%, patients 3, 5 and 6).

**Figure 1:**
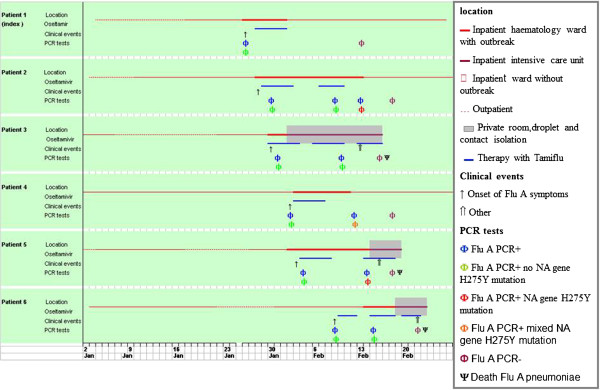
Timeline of influenza A (H1N1) nosocomial during seasonal outbreak: all 6 patients were admitted to the haematology ward.

The clinical characteristics and timeline of the outbreak of H1N1pdm09 in these patients are shown in Table 1 and Figure 1, respectively. The mean age of case patients was 53 (range, 43-65 years) (Table 1). During the seasonal influenza peak all these patients were admitted for reasons unrelated to influenza infection. All patients showed ILI symptoms such as fever, cough, dyspnoea and sore throat, from January 26th to February 14th (Figure 1).

**Table 1:** Clinical Characteristic of six patients with/or without Oseltamivir resistant influenza A (H1N1) virus infection

	**Patient 1 Index case**	**Patient 2**	**Patient 3 Ψ**	**Patient 4**	**Patient 5 Ψ**	**Patient 6 Ψ**
Sex	Female	Male	Male	Male	Male	Male
Age	76	50	39	42	65	24
Medical history	Non Hodgkin Lymphoma	Diffuse large cell Lymphoma	Non Hodgkin Lymphoma, HIV+ patient	Acute Myeloid Leukemia	Multiple myeloma, allogenic stem cell transplantation	Acute lymphoblastic leukemia
Respiratory symptoms	Fever and cough	Fever, Dyspnea, oxygen dependent	Fever, Dyspnea, oxygen dependent	Fever and cough	Fever, Dyspnea, oxygen dependent	Fever, Dyspnea, oxygen dependent
Confirmed diagnosis	Flu lower respiratory tract infection	Pneumonia	Pneumonia	Flu lower respiratory tract infection	Pneumonia	Pneumonia
First positive test result for Flu A	January 26	January 31	January 31	February 1	February 2	February 14
Antiviral theraphy	Oseltamir	Oseltamir	Oseltamir	Oseltamir	Oseltamir	Oseltamir
Oseltamivir resistance	No	Yes	No	Yes	Yes	No
Chest X-ray	No pulmonary abnormalities	Left lower-lobe consolidation	Bilateral lower lobe consolidations	No Pulmonary abnormalities	Perihilar consolidations	Bilateral lower lobe consolidations
Flu A outcome	Viral clearance	Viral clearance	Fatal influenza pneumonia	Viral clearance	Fatal influenza pneumonia	Fatal influenza pnemonia

The presumed index case (patient1) was admitted to the hospital on January 16th for scheduled induction chemotherapy. She was the first case to exhibit symptoms of influenza. Oseltamivir administration was initiated after H1N1pdm09 detection by RT- PCR and lasted for five days (75 mg twice per day). No other viral and bacterial respiratory pathogens were detected, and blood cultures remained negative. At the same time, the screening for all patients started. Four underlying malignancies patients (patient 2, 3, 4 and 5) developed hospital-acquired influenza A virus infection (Table 1) and were present at the same time as the index patient at the Hematology ward, without isolation procedures (Figure 1). Patient 4 developed mild influenza symptoms with rapid viral clearance, patient 2 developed pneumonia but with resolution, whereas patients 3 and 5 developed pulmonary consolidations and fatal influenza pneumonia.

Patients 6 was an ambulatory patient of the Hematology ward, present in the unit at the same time of outbreak for routine analysis. He developed pulmonary consolidation and fatal respiratory failure. The influenza vaccination status of these patients is not known.

All subjects were ambulatory patients, and periodically left the ward and interacted with staff, visitors and other inpatients while outside of their rooms. All patients resulted positive to H1N1pdm09 virus and were administered oseltamivir therapy: 75 mg twice/ day, for five days.

Patients 2, 3, 5, 6 were administered oseltamivir therapy several times and OST-r H1N1pdm09 with NA gene H275Y mutation, as detected by RT PCR and confirmed by Sanger sequencing method, emerged in patients 2, 4 and 5 (3/23, 13.1%). Mixtures of wild-type and mutant variant (H275 H/Y) were detected only in the respiratory specimens from patient 4. Three patients had notable co-pathogens identified. Patients 3 and 6 had concurrent herpesviridae (cytomegalovirus and EBV) detected by RT-PCR in respiratory specimens and blood. Patients 4 had Stafilococcus epidermidis bacteraemia from an unrelated source.

Three patients (3, 5, and 6) were admitted to medical ICU, they had radiologically confirmed pneumonia with unilateral/bilateral opacities. Findings on chest radiographs were consistent with an acute respiratory distress syndrome that required intubation and mechanical ventilation. They died of fatal influenza pneumonia. Remarkably, only one of the three (patient 5) was infected by OST-r H1N1pdm09 variant. Death cause for fatal influenza pneumonia was supported by detection of H1N1pdm09 from post-mortem pulmonary tissue. Among the remaining 17 infected inpatients, 12 patients developed influenza lower respiratory tract infection with different levels of respiratory failure, despite the oseltamivir therapy (diagnosis of H1N1pdm09 was confirmed by NAT of bronchial washings).

Moreover, it is interesting to note that, in this cluster of patients, viral shedding persisted for a very long period (median: 12 days, range: 7-25), longer than the time of therapy which was administered according to guideline (5 days).

## Discussion

We report a case series of hospitalized hematologic/oncologic patients with H1N1pdm09 infection during the seasonal influenza period 2010-2011 in the Hematology ward at Spedali Civili of Brescia. In this period the Hematology division was crowded with patients and when the first case of H1N1pdm09 was identified, the nosocomial influenza virus outbreak spread. Unfortunately, isolation of the first infected patients was not possible.

76 patients developed ILI, but only 23 (30.2%) were infected of H1N1pdm09. Following a confirmed close contact, with index patient, the other patients (53), were infected by other respiratory viruses and bacterial pathogens, which were detected by routine diagnostic procedures by RT-PCR. We are unable to establish whether the health care workers or visitors contributed to viral transmission among the patients, but during that time of year the circulation of H1N1pdm09 was significantly high in the community (93% of Influenza A-positive cases were due to H1N1pdm09) and in our hospital the total H1N1pdm09 infected patients were 57 (15%) out of 379 ILI patients. A rate of 91% of nosocomial patients with Influenza A virus illness was H1N1pdm09 infected and 9% was infected by influenza A (H3N2) or unsubtypable influenza A and influenza B.

The 57 H1N1pdm09 infected patients were subjected to drug-resistance test analysis and five resulted OST-r. These outcomes are similar to the results obtained from other Italian authors [15-17].

Many factors have added up to nosocomial spread of influenza: temporal overlap of inpatient stay within the ward, prolonged viral shedding and presence of health care workers and family members with influenza-like illness. Patients with hematologic malignancies accounted for 50% of deaths among the patients with H1N1pdm09 infection in our Hospital (six patients died: 3 in Hematology ward, 2 in Infectious Diseases division, 1 in Pneumology unit). The mortality rate of RT-PCR confirmmed H1N1pdm09 infected patients in our Hospital was to 10.5%, while in Hematology ward it was 13.1%. These patients were admitted to the intensive care unit due to development of severe respiratory failure.

In addition, our investigation confirmed that a cluster of oseltamivir resistant H1N1pdm09 infection occurred among 3 immuno-compromised patients in the Hematology ward after therapy with oseltamivir. One of them died in ICU and developed OST-r after several weeks of therapy. No evidence was found that health care personnel or other inpatient contacts developed ILI caused by OST-r H1N1pdm09 variant. Resistance to oseltamivir in H1N1pdm09 has been linked to H275Y mutation in NA glycoprotein gene [25-27] and OST-r H1N1pdm09 variant has been described in June 2009 among immuno-compromised patients treated with oseltamivir [2,4,18,28] and OST-r H1N1 2009 virus was first reported in September 2009 [2,28,29] and among fit people who received oseltamivir therapy. Oseltamivir resistance has been detected in less than 1% of untreated patients in the community, and transmission has been documented only in closed settings or settings involving close contact with infected people [22,30]. Moreover, the prevalence of OST-r viruses in the H1N1pdm09 was very low because they are characterized by lower virulence than that of wild-type viruses [31,32], and most fatal cases of OST-r virus infection have occurred in patients with severe immune-suppression associated with hematologic disorders [33,34] or individual risk factors as obesity or diabetes [15,17]. However, current widespread circulation of OST-r pandemic influenza A (H1N1) 2009 virus associated with typical influenza illnesses and viral pneumonia suggest that these viruses retain significant transmissibility and pathogenicity [33-36].

Our analysis of a critical cluster of patients with H1N1pdm09 infection reveals that this disease affected middle age patients, most of them with underlying chronical diseases. Indeed, we observed that patient 5 who became infected by drug resistant virus after prolonged therapy, developed pneumonia, rapidly progressing and leading to death. Other investigators reported similar results: usually, resistance in these patients emerges in response to antiviral drug selection pressure [4,16,18,20-22].

We found a relatively high incidence of drug resistant viruses, i.e. 3 out of 23 H1N1pdm09 infected patients, as confirmed by RT-PCR, after several cycles of oseltamivir therapy. This also appeared to affect the severity of the disease, as these three positive patients were critically ill. The OST-r in hematologic/oncological patients suggests that immuno-suppressed patients have increased risk for complications of influenza and of developing resistant strains [33,34,36]. Early identification and prolonged isolation precautions appear necessary in taking care of infected immuno-compromised patients like critical hematologic/oncological patients. For prevention and control of seasonal influenza among these patients, influenza vaccination is recommended, (as well as in their family members and health care workers), although the immune response to vaccination can be low in these patients [31,32,35].

## Conclusions

Our study has limitations since it is a retrospective, single center observational study, with a relatively small set of data analyzed. The pathogenetic role of OST-r variants observed remains to be confirmed in a larger cohort. Nevertheless, our results underscore the need for diagnostic vigilance and early isolation to prevent nosocomial transmission of influenza and other seasonal influenza infections among immuno-compromised patients. Moreover, influenza virus and other respiratory viruses should always be considered in the differential diagnosis of fever or respiratory symptoms in hospitalized patients when the prevalence of such respiratory viral infections is high in the community.

## Competing interests

All authors declare that they have no competing interests.

## Authors’ contributions

All authors made substantive intellectual contributions to this study. PC, PG, CB, PF contributed significantly to the study conception and design. PC, PG, CS, DTD, PF contributed significantly to the data analysis and interpretation. RG and CC were responsible for the patient enrolment and the data collections. CB supervised to the manuscript draft. All authors read, commented on and approved the final manuscript version.

## Pre-publication history

The pre-publication history for this paper can be accessed here:

http://www.biomedcentral.com/1471-2334/13/127/prepub
